# Editorial: Computational drug discovery for emerging viral infections

**DOI:** 10.3389/fmicb.2023.1326731

**Published:** 2023-11-20

**Authors:** Sinosh Skariyachan, Kumar Kalavathi Murugan, Arli Aditya Parikesit

**Affiliations:** ^1^Department of Microbiology, St. Pius X College Rajapuram, Kasaragod, Kerala, India; ^2^Department of Bioinformatics, Pondicherry University, Puducherry, Tamil Nadu, India; ^3^Department of Bioinformatics, School of Life Sciences, Indonesia International Institute for Life-Sciences (i3L), Jakarta, Indonesia

**Keywords:** emerging viral infections, drug discovery, data science, computational biology, bioinformatics, integrated omics, novel insights, cutting-edge research

The emerging and remerging viral infections mainly SARS-CoV2, Nipah, Zika, Lassa, Monkeypox (mpox), West Nile, and Ebola, are significant concerns for public health in recent the past with high mortality and morbidity throughout the world. Though vaccines have been developed for some of these infections, the lack of promising lead and drug candidates against many of these emerging viruses is a present concern (Morens and Fauci, 2020). Screening of potential molecular targets and unexplored lead molecules from natural sources and repurposing of drug molecules are recent trends in drug discovery. Computational Biology and Bioinformatics potentially contribute to the modern drug discovery protocol (Zhang et al., 2022). The integration of data science, machine learning, and artificial intelligence with computational biology offers great scope in screening lead candidates with ideal drug likeliness, pharmacokinetic and toxicities properties for drug development (Rai et al., 2023). The computational models not only provide insights into the experimental validation, but also reduce the cost, time and other complexities associated with traditional drug discovery and development. Hence, with the advent of molecular docking and dynamics method, computational research is becoming more important than ever for drug development against viral infections (Karplus and McCammon, 2002; Shoichet et al., 2002).

This Research Topic aimed to provide a recent outline and update on various aspects of drug discovery for emerging viral infections that utilize data science, computational biology, bioinformatics, and integrated omics sciences. The major goal of this Research Topic is to prioritize cutting-edge *in silico* drug discovery approaches that can be scaled up to the experimental level and contribute to modern drug discovery against emerging viral infections.

The Research Topic was introduced in May 2022, within the next 6 months we received 23 submissions, of which 12 full-length research articles were accepted and 11 manuscripts were rejected. We have contacted 100% of the potential authors and 100 authors contributed to the Research Topic. As of the second week of October 2023, 19 K total views and downloads were reported. The accepted manuscript contributes substantial breakthroughs in the scope and applications of computational drug discovery and predictive models in future-generation drug discovery to emerging viral infections. Though the Research Topic welcomed the submissions related to emerging viral infections, most of the articles were focused on the role of computational biology and other *in silico* approaches against SARS-CoV-2.

Khan and Lee comprehensively reported the network pharmacological and molecular docking-based studies of using the phytochemicals of *Kochiae fructus* toward the potential molecular targets for the lead discovery against SARS-COV2. The molecular interaction and binding stability of small molecule N-0385 toward TMPRSS2, ACE2, and DPP4 receptors of SARS-CoV-2 were reported (Cao et al.). Han et al. also identified five potential natural compounds against the S protein of Lambda and Delta mutants of SARS-CoV-2. Similarly, with the help of structure-based virtual screening and deep learning approaches, potential ligands were screened toward the main proteases of SARS-CoV-2 (Prabhakaran et al.). The common pathogenic processes between SARS-COV2 and Hemorrhagic fever with renal syndrome (HFRS) are well illustrated by the integration of RNA-seq differential expression analysis and machine learning approaches (Noor et al.). Further, machine learning approaches were used for the large-scale screening for SARS-CoV-2 host factories and prioritized PRI-724 as a potent antiviral against β-catenin/CBP (Kelch et al.). The specific role of immunogenic cell death (ICD) and related genes in SARS-COV2 was analyzed by Zhuo et al. they suggested that *CASP1, CD4*, and *EIF2AK3* are probably the diagnostic genes of SARS-COV2 and that are correlated with immune activity.

In addition to SARS-COV2, the topic collection also highlighted the role of various computational biology approaches and models toward the drug discovery against Lassa virus, West Nile virus, Monkeypox virus and tick-borne pathogens such as human *Babesia microti*. Rcheulishvili et al. designed a universal multiepitope DNA vaccine candidate against mpox and its efficiency was evaluated by immunoinformatic approaches. Several natural plant-based molecules were subjected to computational screening and potential lead molecules were identified as the probable molecular targets of *Babesia microti* and monkeypox virus (Akash, Ahmad Mir et al.). The scope of Xuanbai Chengqi decoction (XBCQD) on monkeypox is illustrated by several computational biology resources by Jioa et al. suggested that the estrone-target AR interaction model is probably one of the best models against monkeypox drug discovery. The modified evodiamine derivatives are probably promising lead candidate against the glycoprotein and nucleoprotein targets of the Lassa virus (Akash, Baeza et al.). Akash, Bayil et al. also performed the interaction modeling of envelope glycoprotein and methyltransferase of West Nile virus and phytocompounds using several *in silico* approaches.

The major focus of the Research Topics is *Computational drug discovery for emerging viral infections*, the authors comprehensively illustrated the scope and applications of various predictive models in drug screening and new lead discovery against various emerging infections such as SARS-CoV2, Monkeypox and Lasa Viral infections, the computational models require extensive studies and validation in experimental level. Computational drug discovery certainly reduces the time, complexities, and initial investments associated with conventional vaccine design and drug discovery. The predictive models provide substantial breakthroughs and a foundation to scale up the hypothetical aspects into the experimental level and can scale up the approach into the industrial and healthcare sectors.

## Author contributions

SS: Conceptualization, Data curation, Formal analysis, Methodology, Resources, Validation, Writing—original draft. KK: Investigation, Project administration, Supervision, Visualization, Writing—review & editing. AP: Investigation, Project administration, Resources, Supervision, Writing—review & editing.
